# Supplementary material for: Multiple serine transposase dimers assemble the transposon-end synaptic complex during IS607-family transposition
Source: eLife. 2018 Oct 5;7:e39611. doi: 10.7554/eLife.39611 (PMC6188088; doi:10.7554/eLife.39611)
Supplement: Supplementary file 1. [file elife-39611-supp1.docx]

**Supplementary file 1.** *E. coli* strains used in this work.

| Strain | Description | Source |
| --- | --- | --- |
| RJ1224 | *recA56, srl,* Δ*(pro-lac), ara, rpsL*, λ*bbnin* [λ *cI857, b515, b519, nin5, S_am_7*] | Laboratory collection |
| Hfl-1 | *hfl-1,* *fhuA2::IS2*, *lacY1*, *tsx-1*, *glnX44*, *gal-6*, *xyl-7*, *mtlA2*, *mut-14* | (Belfort and Wulff 1973) |
| LE392 | *hsd*R514 (rk^–^, mk^+^), *gln*X (supE44), *tryT (supF58), Δ(codB-lacI)3, galK2, galT22, metB1, trpR55* | W. Reznikoff |
| BW14879 | pMW11 Muc62 *Δ(lac)X74, Δ(phoA532 Pvull) phn (EcoB), arcA1655, fnr-1655* | B. Wanner [(Metcalf](http://www.ncbi.nlm.nih.gov/pubmed/2160940) et al. 1990) |
| BW5104 | Mu-1 *Δlac169, creB510, hsdR514* | B. Wanner (Metcalf et al. 1990) |
| RJ3960 | BW5104 λ^R^ *mal* | This study |
| RJ3388 | BL21 (DE3) *endA::tet8, fis::str/spc-985* | Laboratory collection |
| RJ3431 | BL21 (DE3) *metC::Tn10* | Laboratory collection |
