# Supplementary material for: Multiple serine transposase dimers assemble the transposon-end synaptic complex during IS607-family transposition
Source: eLife. 2018 Oct 5;7:e39611. doi: 10.7554/eLife.39611 (PMC6188088; doi:10.7554/eLife.39611)
Supplement: Supplementary file 2. [file elife-39611-supp2.docx]

**Supplementary file 2.** Plasmids used and constructed in this work.

| Plasmid | Description* | Source/construction |
| --- | --- | --- |
| pRZ104 | colE1-Tn*5* | W. Reznikoff (Jorgensen et al. 1979) |
| pMW11 | miniMu cloning vector, *str/spc* | B. Wanner (Metcalf et al. 1990) |
| pRJ3346 | pET11a - IS*1535 tnpA^6His^* | oRJ801+oRJ802 on *M. tuberculosis* gDNA; into pET11a (NdeI-BamHI) |
| pRJ3347 | pET21a - IS*1535 tnpA (aa51-193)^6His^* | oRJ803+oRJ804 on pRJ3346: into pET21a (NdeI-SalI) |
| pRJ3275 | pET11a - IS*1535 tnpA (aa1-146)^6His^* | oRJ801+oRJ805 on pRJ3346; into pET11a (NdeI-BamH1) |
| pRJ3364 | pET32a - *malE* | oRJ806+oRJ807 on pMAL-c2 (NEB); into pET32a (BamHI-XhoI) |
| pRJ3327 | pET32a - IS*1535 tnpA-malE fusion^6His^* | oRJ801+oRJ808 on pRJ3346; into pRJ3364 (NdeI-BamH1) |
| pRJ3277 | pET11a - IS*1535 tnpA(F126C)^6His^* | QuikChange: oRJ809+ oRJ810 on pRJ3346 |
| pRJ3280 | pET11a - IS*1535 tnpA(Q138C)^6His^* | QuikChange: oRJ811+ oRJ812 on pRJ3346 |
| pRJ3278 | pET11a - IS*1535 tnpA(L162C)^6His^* | QuikChange: oRJ813+ oRJ814 on pRJ3346 |
| pRJ3276 | pET11a - IS*1535 tnpA(A182C)^6His^* | QuikChange: oRJ815+ oRJ816 on pRJ3346 |
| pRJ3289 | pET11a - IS*1535 tnpA(C90S/C156S/C166S)^6His^* | Sequential QuikChange on pRJ3346: oRJ817+oRJ818, oRJ819+oRJ820, oRJ821+oRJ822 |
| pRJ3293 | pET11a - IS*1535 tnpA(C90S/C156S/C166S/F126C)^6His^* | QuikChange: oRJ809+oRJ810 on pRJ3289 |
| pRJ3292 | pET11a - IS*1535 tnpA(C90S/C156S/C166S/Q138C)^6His^* | QuikChange: oRJ811+oRJ812 on pRJ3289 |
| pRJ3298 | pET11a - IS*1535 tnpA(C90S/C156S/C166S/Q162C)^6His^* | QuikChange: oRJ813+oRJ814 on pRJ3289 |
| pRJ3253 | pUC18 – ISC1926 | oRJ823+oRJ824 on *S. islandicus* *pyrE*::ISC1926 gDNA; into pUC18 (SmaI) |
| pRJ3248 | pET21a-ISC*1926 tnpA^6His^* | oRJ825+oRJ826 on pRJ3253; into pET21a (NdeI-XhoI) |
| pRJ3363 | pET21a-ISC*1926 tnpA (aa 65-211)^(N)6His^* | oRJ827+oRJ828 on pRJ3248 into pET21a |
| pRJ3260 | pUC57-IS*607* *tnpA* | *orfA* (sequence below, Genewiz) in pUC57 |
| pRJ3297 | pET11a-IS*607* *orfB* | *orfB* (sequence below, Genewiz) in pET11a (NdeI-BamHI) |
| pRJ3261 | pUC18-IS*607* left end (LE) | LE (sequence below) into pUC18 (SmaI) |
| pRJ3262 | pUC18-IS*607* right end (RE) | RE (sequence below) into pUC18 (SmaI) |
| pRJ3264 | pACYC184-*lacPO* | 204 bp *lacPO* into pACYC184 (AvaI-HindIII) |
| pRJ3268 | pRJ3264-*P_lac_*-*IS607-tnpA* | Subclone from pRJ3260 (NdeI+BamHI) |
| pRJ3310 | pRJ3264-*P_lac_*-*IS607-tnpA-rbs-orfB* | oRJ829+oRJ830 on pRJ3297; into pRJ3264 (BamHI-HindIII) |
| pRJ3321 | pBR322-IS*607 (LE-tet-RE)* | oRJ831+oRJ832 on pRJ3261; into pBR322 (AatII-EcoR1), oRJ833+oRJ834 on pRJ3262; into pBR322 (StyI-Ava1) |
| pRJ3330 | pBR322-IS*607 (LE-P_lac_-tnpA-tet-RE)* | oRJ835+oRJ836 on pRJ3268; into pRJ3321 (EcoR1) |
| pRJ3332 | pBR322-IS*607 (LE-P_lac_-tnpA-orfB-tet-RE)* | oRJ835+oRJ836 on pRJ3310; into pRJ3321 (EcoR1) |
| pRJ3338 | pBR322-IS*607 (LE-P_lac_-tnpA(S72G)-tet-RE)* | QuikChange: oRJ837+oRJ838 on pRJ3330 |
| pRJ3234 | pUC18-IS*1535* left end | oRJ839+oRJ840 on *M. tuberculosis* gDNA; into pUC18 (SmaI) |
| pRJ3348 | pUC18-IS*1535* right end | oRJ841+oRJ842 on *M. tuberculosis* gDNA; into pUC18 (SmaI) |
| pRJ3350 | pUC18-IS*1535 LE: 20-69* | Anneal oRJ843+oRJ844; into pUC18 (SmaI) |
| pRJ3351 | pUC18-IS*1535 LE: 20-64* | Anneal oRJ845+oRJ846; into pUC18 (KpnI-PstI) |
| pRJ3352 | pUC18-IS*1535 LE:20-54* | Anneal oRJ847+oRJ848; into pUC18 (KpnI-PstI) |
| pRJ3355 | pUC18-IS*1535 LE:20-49* | Anneal oRJ849+oRJ850; into pUC18 (KpnI-PstI) |
| pRJ3353 | pUC18-IS*1535 LE:20-44* | Anneal oRJ851+oRJ852; into pUC18 (KpnI-PstI) |
| pRJ3354 | pUC18-IS*1535 LE:20-39* | Anneal oRJ853+oRJ854; into pUC18 (KpnI-PstI) |

* Where designated, His tags are at the C-terminal end of the protein unless noted as (N).

IS*607* *orfA/tnpA* sequence:

ATGAACAAGCGTATGCTGAGCATTGGCCAGGCCAGCAAACTGCTGGGTGTGACCATCCAGACCCTGCGCAACTGGGACAAAAAGGACCTGCTGAAACCGGATGAGCTGACCAAAGGCGGTGAACGCCGTTATAAACTGGAAAGCCTGCGCCGCATCAACCGCAGCATTGTGTTCAACCAGGACGAGCTGAAGACCATTGCCTACGCCCGCGTGAGTAGCCATGATCAGCAGGATGATCTGATCCGTCAGGTTCAGGTTCTGGAGCTGTACTGCGCCCGCTGTGGCTTTAACTATGAAGTGATTCAGGATCTGGGCAGCGGTATGAACTACTACAAGAAGGGCCTGACCAAGCTGCTGAATCTGATCCTGGACAACCAGGTGAAGCGCCTGGTGCTGACCCATAAAGATCGTCTGCTGCGCTTCGGCGCAGAACTGGTGTTTAGCATTTGCGAAGCCAAGGGTGTGGAGGTGGTGATCATCAACAAAGGCGACGAGAACGTGCGCTTTGAAGAGGAGCTGGCCAAGGATGTTCTGGAGATTATCACCGTGTTTAGTGCCCGCCTGTACGGTAGCCGCAGCAAGAAAAATAAAAAACTGCTGGACGAGATGCAAGAAGTTATCACCAACAACGTGAGTTACCTGAATCATGCCTAA

IS*607* *orfB* sequence:

ATGAGCGCGATTAGCATCACCCATAAGATTGCGCTGAAGCCGAACAACAAGCACATTACCTATTTTAAGAAGGCGTTTGGTTGCGCGCGTTTCGCGTACAACTGGGGTCTGGCGAAATGGAAGGAGAACTATCAGCTGGGCATTAAGACCAGCCACCTGCAACTGAAGAAAGAGTTTAACGCGCTGAAGAAAAGCCAGTTCAACTTTGTGTACGAAGTTACCAAATATGCGACCCAGCAACCGTTCATCCACCTGAACCTGGCGTTTAACAAGTTCTTTCGTGACCTGGAGAAAGGTCTGGTGAGCTACCCGAAGTTCAAGAAAAAGCGTGAGTTCCAAGGCAGCTTTTATATTGGTGGCGACCAGATCAAAATCATTCAAACCGCGAACACCGATTACCTGAAGATTCCGAACCTGCCGCCGATCAAACTGACCGAGAAGCTGCGTTTCCAGGGTAAAATTCACAACGCGACCATCACCCAAAAGGGCGATCACTTTTACGTTAGCATTAGCTGCGACATCGATGAGAGCGAATATAAACGTACCCACAAGCTGCAGGAAAGCCACAACAAACTGGGTATCGACATTGGCATCAAGAGCTTCGTGAGCCTGAGCAACGGTCTGAACATTTACGCGCCGAAACCGCTGGATAAGCTGACCCGTAAACTGGTTCGTATTAGCCGTCAACTGAGCAAAAAGATCCACCCGAAAACCAAGGGCGACAAAACCCGTAAGAGCAACAACTATCTGAAACACAGCAAAAAGCTGACCCACCTGCACGAGAAGATTGCGAACATCCGTCTGGATTTCCTGCACAAACTGACCAGCAGCCTGATCCGTCACAGCAACAGCTTCTGCCTGGAAAGCCTGAAAGTGAAGAACATGTTTAAGAACCACCGTCTGGCGAAAAGCCTGAGCGACATTAGCATGAGCGTTTTTAACACCCTGCTGGAGTACAAAGCGAAGTATAGCAACAAGGAAATCCTGCGTGCGGATACCTACTATCCGAGCAGCAAAACCTGCAGCAACTGCCAGAAAGTGAAGCAAGACCTGAAACTGAAGGATCGTATCTACCAGTGCCTGGAGTGCGGTTTCGAACTGGACCGTGATATTAACGCGGCGATCAACCTGCTGAAGCACCTGGTGGGCCGTGTTACCGCGGAATTTACCCCGATGGACCTGACCGCGCTGCTGAACGATCTGAGCAACAATCGTCTGGCGACCAGCAAAGTGGAACTGGGCATTCAACAAAAGAGCTAA

IS*607* left end + host (underlined) sequence:

ATTGATAGCATTCTTTTATTCATGCAAGCAGTATAACACAAAACTTAATAACTTATACAAAAATTATAGTATTTTATAGGTTATTATAGGATTTTAGTTTCTGTTTGTAGCCCATAACGGCTACACTTCTAATC

IS*607* right end + host (underlined) sequence:

TATCCAATAATCGTTTAGCAACTAGCAAGGTTGAACTAGGAATACAACAAAAATCCTAAATTAAGAGAATTTTATAGCTCTTTATAGGATTTTATAGGTTTGTAGTAACGGTGTTAATAACCCACAAATCCGC
