# Supplementary material for: Multiple serine transposase dimers assemble the transposon-end synaptic complex during IS607-family transposition
Source: eLife. 2018 Oct 5;7:e39611. doi: 10.7554/eLife.39611 (PMC6188088; doi:10.7554/eLife.39611)
Supplement: Supplementary file 3. [file elife-39611-supp3.docx]

**Supplementary file 3.** Oligonucleotides used in this work.

| Oligo number | Sequence | Purpose |
| --- | --- | --- |
| oRJ801 | AAAAACATATGAATCTGGCGGATTGGGC | forward primer to amplify IS*1535* *tnpA* gene |
| oRJ802 | AAAAAGGATCCCTAGTGGTGATGGTGATGATGCCCCGCCCCCGGCTCACG | reverse primer to amplify IS*1535 tnpA* gene |
| oRJ803 | AAAACATATGGGAGTGGTGCTGTATGCGCGG | forward primer to amplify the IS*1535 tpnA* CTD |
| oRJ804 | AAAAGTCGACCCCCGCCCCCGGCTCACGC | reverse primer to amplify the IS*1535* *tpnA* CTD gene |
| oRJ805 | ATATATGGATCCTTACTAGTGGTGATGGTGATGATGATCGGCGACCACAATCC | reverse primer to amplify the IS*1535* *tpnA* deletion (aa1-146) with C-terminal His_6_ tag |
| oRJ806 | ATATATGGATCCGGTGCGAGCATGAAAATCGAAGAAGGTAAACTGG | forward primer to amplify *malE* gene from pMAL c2 |
| oRJ807 | ATATATCTCGAGGTGGTGGTGGTGGTGGTGCCCGAGGTTGTTGTTATTGTTATT | reverse primer to amplify *malE* gene from pMAL c2 with a C-terminal His_6_ tag |
| oRJ808 | ATATGGATCCCGCGCTCCCCGCCCCCGGCTCAC | reverse oligo to amplify IS*1535 tnpA* gene |
| oRJ809 | CGGCTGGCGCGTTGCGGGGTGGAGCACC | to generate F126C mutant of IS*1535 tnpA* |
| oRJ810 | GGTGCTCCACCCCGCAACGCGCCAGCCG | to generate F126C mutant of IS*1535 tnpA* |
| oRJ811 | GGCGGCGCTGTCTGCTTGCGGCCGGCGGATTGTGG | to generate Q138C mutant of IS*1535 tnpA* |
| oRJ812 | CCACAATCCGCCGGCCGCAAGCAGACAGCGCCGCC | to generate Q138C mutant of IS*1535 tnpA* |
| oRJ813 | CATGATCGAGGTCTGCACCGGTATGTGCGCGCGGC | to generate L162C mutant of IS*1535 tnpA* |
| oRJ814 | GCCGCGCGCACATACCGGTGCAGACCTCGATCATG | to generate L162C mutant of IS*1535 tnpA* |
| oRJ815 | CCGGGCGATGCGTTGCGTCACGGAGGCC | to generate A182C mutant of IS*1535 tnpA* |
| oRJ816 | GGCCTCCGTGACGCAACGCATCGCCCGG | to generate A182C mutant of IS*1535 tnpA* |
| oRJ817 | GGGTGGGGCAAGTGGTGTCTGAGGTCGGTTCCGGCC | to generate C90S mutant of IS*1535 tnpA* |
| oRJ818 | GGCCGGAACCGACCTCAGACACCACTTGCCCCACCC | to generate C90S mutant of IS*1535 tnpA* |
| oRJ819 | GTGAGACGACCGATGATCTGGTGTCTGACATGATCGAGGTCTTGACCGG | to generate C156S mutant of IS*1535 tnpA* |
| oRJ820 | CCGGTCAAGACCTCGATCATGTCAGACACCAGATCATCGGTCGTCTCAC | to generate C156S mutant of IS*1535 tnpA* |
| oRJ821 | GGTCTTGACCGGTATGTCTGCGCGGCTGTACGGGCG | to generate C166S mutant of IS*1535 tnpA* |
| oRJ822 | CGCCCGTACAGCCGCGCAGACATACCGGTCAAGACC | to generate C166S mutant of *IS1535 tnpA* |
| oRJ823 | AAGGGCTGAATCCTTTCTCACG | to PCR amplify ISC  *1926* transposon & clone into pUC18 at SmaI |
| oRJ824 | GTTTGTCCCTACACTTTCATTTCAATC | to PCR amplify ISC*1926* transposon & clone into pUC18 at SmaI |
| oRJ825 | ATATATCATATGCATTTATTATCAACTAATTTTGTGGAGAGAC | to clone ISC*1926* *tnpA* in pET21b with C-terminal His_6_ tag |
| oRJ826 | ATATCTCGAGACCCTTAACTTTATCGTCCTCTCCAC | to clone ISC*1926* *tnpA* in pET21b with C-terminal His_6_ tag |
| oRJ827 | ATATCATATGCACCATCACCATCACCATATAAGGGCAGTAATTTACGCAAGAGTTTC | forward primer to clone the ISC*1926 tnpA* CTD (aa 65-221) with N-His_6_ tag |
| oRJ828 | TATATAGGATCCTTAGGCATGATTCAGGTAACTCACG | reverse primer to clone ISC*1926 TnpA* gene with stop codon |
| oRJ829 | ATATATGGATCCTAAGAAGGAGATATACCATGAGCGCGATTAGCATCA | forward primer to amplify IS*607 tnpB* gene with rbs |
| oRJ830 | TATATAAAGCTTTTAGCTCTTTTGTTGAATGCCC | reverse primer to amplify IS*607 tnpB* gene |
| oRJ831 | ATATATGACGTCGATTAGAAGTGTAGCCGTTATGGG | to PCR amplify IS*607* LE DNA |
| oRJ832 | ATATATGAATTCATTGATAGCATTCTTTTATTCATGCA | to PCR amplify IS*607* LE DNA |
| oRJ833 | ATATATCCTTGGTATCCAATAATCGTTTAGCAACTAGC | to PCR amplify IS*607* RE DNA |
| oRJ834 | ATATATCCCGAGGCGGATTTGTGGGTTATTAACA | to PCR amplify IS*607* RE DNA |
| oRJ835 | ATATATGAATTCATTAATGCAGCTGGCACGA | to amplify the *Plac-tnpA/+tnpB* DNA fragment from pRJ3264 derived plasmids |
| oRJ836 | ATATATGAATTCTGTTTGACAGCTTATCATCGATAAG | to amplify the *Plac-tnpA/+tnpB* DNA fragment from pRJ3264 derived plasmids |
| oRJ837 | GCCTACGCCCGCGTGgcgAGCCATGATCAGCAGG | to construct S72G on IS*607* *tnpA* |
| oRJ838 | CCTGCTGATCATGGCTcgcCACGCGGGCGTAGGC | to construct S72G on IS*607 tnpA* |
| oRJ839 | AAAAAGACGTCCGTGTGTGGTGGGCGCTTGTC | to PCR amplify IS*1535* LE |
| oRJ840 | AAAAAGAATTCCGACTCCGCCCAATCCGCC | to PCR amplify IS*1535* LE |
| oRJ841 | AAAAACCTTGGCCAAAACCCAAACCCCGCAAG | to PCR amplify IS*1535* RE |
| oRJ842 | AAAAACTCGGGGAGCGGCCGATCACCTGATCG | to PCR amplify IS*1535* RE |
| oRJ843 | GTTGAGTGTGTTTTAGTGTGCGTTAGTGTGTTCTAATTGGCGGCGTGAAT | to anneal and construct pUC18-IS*1535* LE:20-69 (pRJ3350) |
| oRJ844 | ATTCACGCCGCCAATTAGAACACACTAACGCACACTAAAACACACTCAAC | to anneal and construct pUC18-IS*1535* LE:20-69 (pRJ3350) |
| oRJ845 | GTGTGTTTTAGTGTGCGTTAGTGTGTTCTAATTGGCGGCGTGAATGTAC | to anneal and construct pUC18*-*IS*1535* LE:20-64 (pRJ3351) |
| oRJ846 | ATTCACGCCGCCAATTAGAACACACTAACGCACACTAAAACACACTGCA | to anneal and construct pUC18-IS*1535* LE:20-64 (pRJ3351) |
| oRJ847 | GTTGAGTGTGTTTTAGTGTGCGTTAGTGTGTTCTAGTAC | to anneal and construct pUC18-IS*1535* LE:20-54 (pRJ3352) |
| oRJ848 | TAGAACACACTAACGCACACTAAAACACACTCAACTGCA | to anneal and construct pUC18-IS*1535* LE:20-54 (pRJ3352) |
| oRJ849 | GTTGAGTGTGTTTTAGTGTGCGTTAGTGTGGTAC | to anneal and construct pUC18-IS*1535* LE:20-49 (pRJ3355) |
| oRJ850 | CACACTAACGCACACTAAAACACACTCAACTGCA | to anneal and construct pUC18-IS*1535* LE:20-49 (pRJ3355) |
| oRJ851 | GTTGAGTGTGTTTTAGTGTGCGTTAGTAC | to anneal and construct pUC18-IS*1535* LE:20-44 (pRJ3353) |
| oRJ852 | TAACGCACACTAAAACACACTCAACTGCA | to anneal and construct pUC18-IS*1535* LE:20-44 (pRJ3353) |
| oRJ853 | GTTGAGTGTGTTTTAGTGTGGTAC | to anneal and construct pUC18-IS*1535* LE:20-39 (pRJ3354) |
| oRJ854 | CACACTAAAACACACTCAACTGCA | to anneal and construct pUC18-IS*1535* LE:20-39 (pRJ3354) |
| oRJ855 | ATATACCCGAGATTAATGCAGCTGGCACGA | to amplify *Plac* from pUC18 |
| oRJ856 | ATATAAAGCTTGATATCGGATCCGTCGACCATATGTGTTTCCTGTGTGAAATTGTTATCC | to amplify *Plac* from pUC18 |
| oRJ857 | AAAAAGGATCCCTACCCCGCCCCCGGCTCACG | reverse primer to amplify IS*1535 tnpA* gene |
| oRJ858 | GATTAGAAGTGTAGCCGTTATGGGCTACAAACAGAAACTAAAATCCTATAATAACCTATAAAATACTATAATTTTTGTATAAGTTATTAAGTTTTGTGTTATACTGCTTGCATGAATAAAAGAATGCTATCAAT | IS*607* left end sequence to clone into pUC18 at SmaI |
| oRJ859 | TATCCAATAATCGTTTAGCAACTAGCAAGGTTGAACTAGGAATACAACAAAAATCCTAAATTAAGAGAATTTTATAGCTCTTTATAGGATTTTATAGGTTTGTAGTAACGGTGTTAATAACCCACAAATCCGC | IS*607* right end sequence to clone into pUC18 at SmaI |
| oRJ860 | GTAAAACGACGGCCAGTGC | to amplify IS*1535* LE HΔ sequences with oRJ861, oRJ862, oRJ863, oRJ864, oRJ865, oRJ866, oRJ867, or oRJ868 using pRJ3234 as template |
| oRJ861 | GCTCTGGGGTTCAGAGCTG | to amplify IS*1535* LE H6Δ sequence |
| oRJ862 | GGGGTTCAGAGCTGTTGC | to amplify IS*1535* LE H1Δ sequence |
| oRJ863 | TCAGAGCTGTTGCGTGTTG | to amplify IS*1535* LE 5Δ sequence |
| oRJ864 | GCTGTTGCGTGTTGAGTGTG | to amplify *IS*1535 LE 10Δ sequence |
| oRJ865 | GTTGAGTGTGTTTTAGTGTGCGTT | to amplify IS*1535* LE 20Δ sequence |
| oRJ866 | GTGTGTTTTAGTGTGCGTTAGTGTG | to amplify IS*1535* LE 25Δ sequence |
| oRJ867 | AGGGTTTTCCCAGTCACGACGTTGTTGAGTGTGTTTTAGTGTGCGTT | to amplify IS*1535* LE 20v sequence |
| oRJ868 | GTAAAACGACGGCCAGTGCGTGTGTTTTAGTGTGCGTTAGTGTG | to amplify IS*1535* LE 25v sequence |
| oRJ869 | CAGGAAACAGCTATGACATGATTAC | to amplify IS*1535* LE HΔ sequences with oRJ870, oRJ871, oRJ872, oRJ873 or oRJ874, oRJ875 using pRJ3234 as template |
| oRJ870 | GACTCCGCCCAATCCG | to amplify IS*1535* LE Δ89 sequence |
| oRJ871 | CGCCCAATCCGCCAGA | to amplify IS*1535* LE Δ84 sequence |
| oRJ872 | AATCCGCCAGATTCACGC | to amplify IS*1535* LE Δ79 sequence |
| oRJ873 | GCCAGATTCACGCCGC | to amplify IS*1535* LE Δ74 sequence |
| oRJ874 | ATTCACGCCGCCAATTAG | to amplify IS*1535* LE Δ69 sequence |
| oRJ875 | CGCCGCCAATTAGAACAC | to amplify IS*1535* LE Δ64 sequence |
| oRJ876 | ATATATGAATTCGTGGTGGGCGGATTGG | to amplify the IS*1535* LE DNA with oRJ877 to determine whether the PEC DNA strands are parallel or anti-parallel |
| oRJ877 | TGTAAAACGACGGCCAGTG | to amplify the IS*1535* LE DNA with oRJ876 to determine whether the PEC DNA strands are parallel or anti-parallel |
| oRJ878 | GCCGGAAGCATAAAGTGTAAAG | to sequence IS*607-tet* insertion sites from left end |
| oRJ879 | GGATTCACCACTCCAAGAATTG | to sequence IS*607-tet* insertion sites from right end |
| oRJ880 | GTAAAACGACGGCCAGT | to amplify IS*1535* footprint probes, bottom strand |
| oRJ881 | CAGGAAACAGCTATGACCA | to amplify IS*1535* footprint probes, top strand |
| oRJ882 | GGGTAACGCCAGGGTTTTC | to amplify the 240 bp IS*1535* probe with oRJ839 |
